# Detailed observation of anatomical location and pattern in Hangman’s fracture based on computed tomography three-dimensional reconstruction

**DOI:** 10.1186/s13018-023-03622-x

**Published:** 2023-02-23

**Authors:** Guangzhou Li, Qing Wang

**Affiliations:** Department of Orthopeadics (Spine Surgery), The Affiliated Hospital of Southwest Medical University, No. 25 Taiping Street, Luzhou, 646000 Sichuan Province China

**Keywords:** Hangman’s fractures, Axis, Fracture lines, Anatomical location, Pattern, Three-dimensional reconstruction

## Abstract

**Objective:**

To observe the precise anatomical location and pattern of the fracture lines in Hangman’s fracture.

**Methods:**

Three-dimensional computed CT images of 210 patients with Hangman’s fracture were collected. According to the involvement of anatomical structures, the injuries were classified into facet joint injury and pure bony injury. The C2 ring was also divided into: anterior, middle, and posterior elements. The anatomical structures involvement and fracture patterns were observed.

**Result:**

Total 520 anatomical structures injuries were involved in 210 patients Hangman’s fractures, including 298 facet joints injuries (57.3%) and 222 bony injuries (42.7%). The most common facet joints injury was superior articular facet injury of C2, and the most common pure bony injury was pediculoisthmic component fracture. The injuries of anterior element (60.6%) were more common than that of middle (20.4%) or posterior (19.0%) element. One injury in anterior element on one side and another injury located in the anterior, middle or posterior element other side was the most common fracture pattern. Injury of middle element on one side with another injury located in the middle or posterior element could be also observed.

**Conclusion:**

In Hangman’s fractures, fracture lines could occur in any part of C2 ring. Facet joints injuries were more common than pure bony injuries, and the injuries of anterior element were also more common than that of middle or posterior element. The high prevalence of facet joints injuries means that most of Hangman’s fractures may be involved with intra-articular injuries.

## Introduction

Hangman’s fracture, also referred to as traumatic spondylolisthesis of the axis or fracture of the axis (C2) ring, is the second most common injury of the axis [11, 21]. It is estimated that Hangman’s fracture accounts for 20–22% of all axis injuries [1, 19]. It was once thought that fractures of bilateral pars or pedicle of C2 were basic features for such injuries, due to early studies investigating the anatomical features mainly basing on X-ray films of cervical spine [3–5, 10]. With the development of imaging technique and the extensive use of computed tomography (CT) scans, it is showed that symmetrical typical bilateral pedicle or par fractures of axis are rare, and fracture lines might occur through any part of C2 ring, including superior or inferior articular processes, pedicle, pars interarticularis, laminae, posterior vertebral wall of C2, and so on [1, 10, 12, 14–16, 21, 22].

Levine–Edwards’ classification was the most widely accepted and used as guide the choice for Hangman’s fracture, and the essence of Levine–Edwards’ classification is that it can evaluate whether C2-3 is unstable according to the displacement and angulation between C2-3 on X-ray films [9, 12]. However, the management of Hangman’s fracture is not without controversial [19]. Apart to considering the integrity of C2-3 discoligamentous structures, an ideal treatment scheme for Hangman’s fracture might be tailored according to the fracture line location, fracture pattern, and even injury mechanism [12, 13, 16]. Therefore, one question needs to be answered: What is the precise anatomical location and pattern of the fracture lines in such fractures based on large or relative large size sample database [16]?

To the best of our knowledge, no studies with a large sample so far address this question. Therefore, the objective of this study was to observe the precise anatomical location and pattern of the fracture lines in Hangman’s fracture by reviewing the clinical and radiological data from prospectively maintained database.

## Materials and methods

### Patients

We reviewed all the medical records in our prospectively maintained database, which accumulated 372 cases with C2 vertebra or axis ring fractures from seven tertiary referral medical centers in China between December 2013 and November 2019. This study project was approved by the institutional research and ethics committee.

Inclusion criteria were: (1) patients were medically confirmed of Hangman’s fractures, which were defined as axis ring fractures with or without displacement and/or angulation at C2/3 level [1, 3, 10, 12]; (2) patients with complete imaging studies, including X-rays, axial plane CT scans, sagittal and coronal plane reconstructions, and three-dimensional reconstructions of cervical spine, and all CT images were acquired using at least a 64-slice multidetector CT scanner [16]. Patients with congenital anomalies, infections, or tumors in the upper cervical spine were excluded [7, 23].

According to the inclusion and exclusion criteria, 210 patients with Hangman’s fractures were included in this study, and 162 patients were excluded, including 83 patients with Hangman’s fractures (61 due to complete imaging studies; 22 due to congenital anomalies, infections, or tumors in the upper cervical spine) and 79 with fracture of C2 vertebral body or lateral mass.

There were 154 male and 56 female, and the average age at injury was 49.3 years (range, 15–91 years). The most common causes of injury were falls, causing 97 (46.2%) injuries. Motor vehicle accidents were second common, accounting for 70 (33.3%) injuries. The less common causes of injury were low energy injuries (e.g., fallen from standing height), causing 27 (12.9%) injuries. The remaining causes of injury were others, causing 16 (7.6%) injuries.

### Radiographic assessment

For the purpose of this review, only pre-treatment imaging studies were collected and analyzed. According to the involvement of anatomical structures, the axis ring injuries were classified into facet joint and pure bony injuries. Facet joint injury included superior or inferior articular facet injuries of the axis, and C2-3 disk injuries, which was defined if MRI of cervical spine showed damage of C2-3 discoligamentous structures or X-ray films showed significant angulation (> 11 degrees) and/or anterior translation (> 3 mm) of C2-3 [1, 9]; Pure bony injury included the posterior vertebral wall fractures, pediculoisthmic component (PIC) fractures [20, 24], and lamina fractures of the axis.

**Fig. 1:**
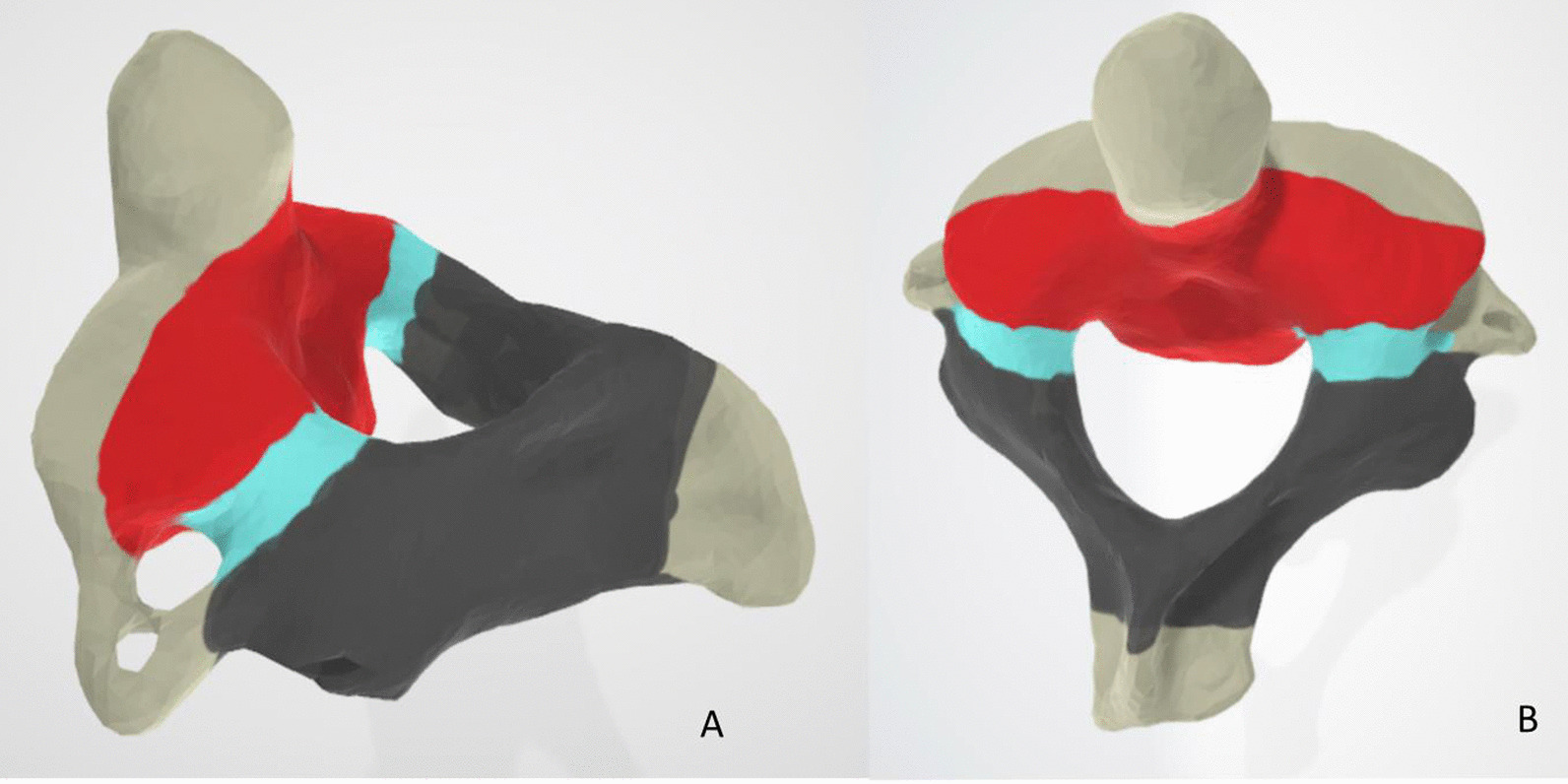
Diagram of C2 ring (**A** left side view; **B** top view) showing anterior (marked by the red area), middle (marked by the acid blue area), and posterior (marked by the black area) elements

The C2 bony ring was divided into three parts: anterior element, which included bilateral superior articular processes and the posterior vertebral wall; middle element, the only component was PIC [20]; and posterior elements, which included bilateral inferior articular processes and lamina (Fig. 1).

**Fig. 2:**
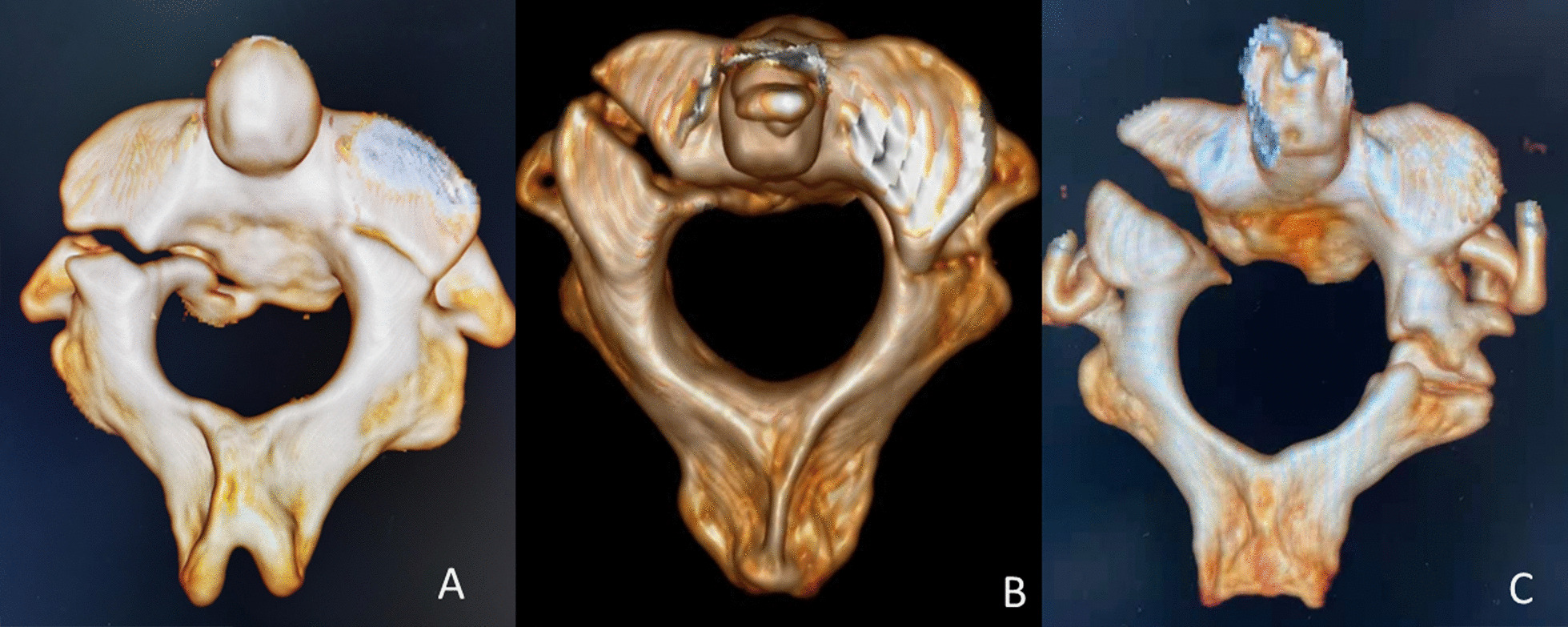
Three-dimensional reconstruction CT image showed injuries of anterior element on one side and the other side injury located in the anterior, middle or posterior element: **A** two anterior elements of C2 ring, with left fracture line extending from superior articular process to posterior vertebral wall and right fracture line through superior articular process; **B** anterior and middle elements injuries, with left fracture line through posterior wall and upper articular surface of C2 and right PIC fracture; **C** anterior and posterior elements injuries with left fracture line through posterior wall and upper articular surface of C2 and right inferior articular process fracture

**Fig. 3:**
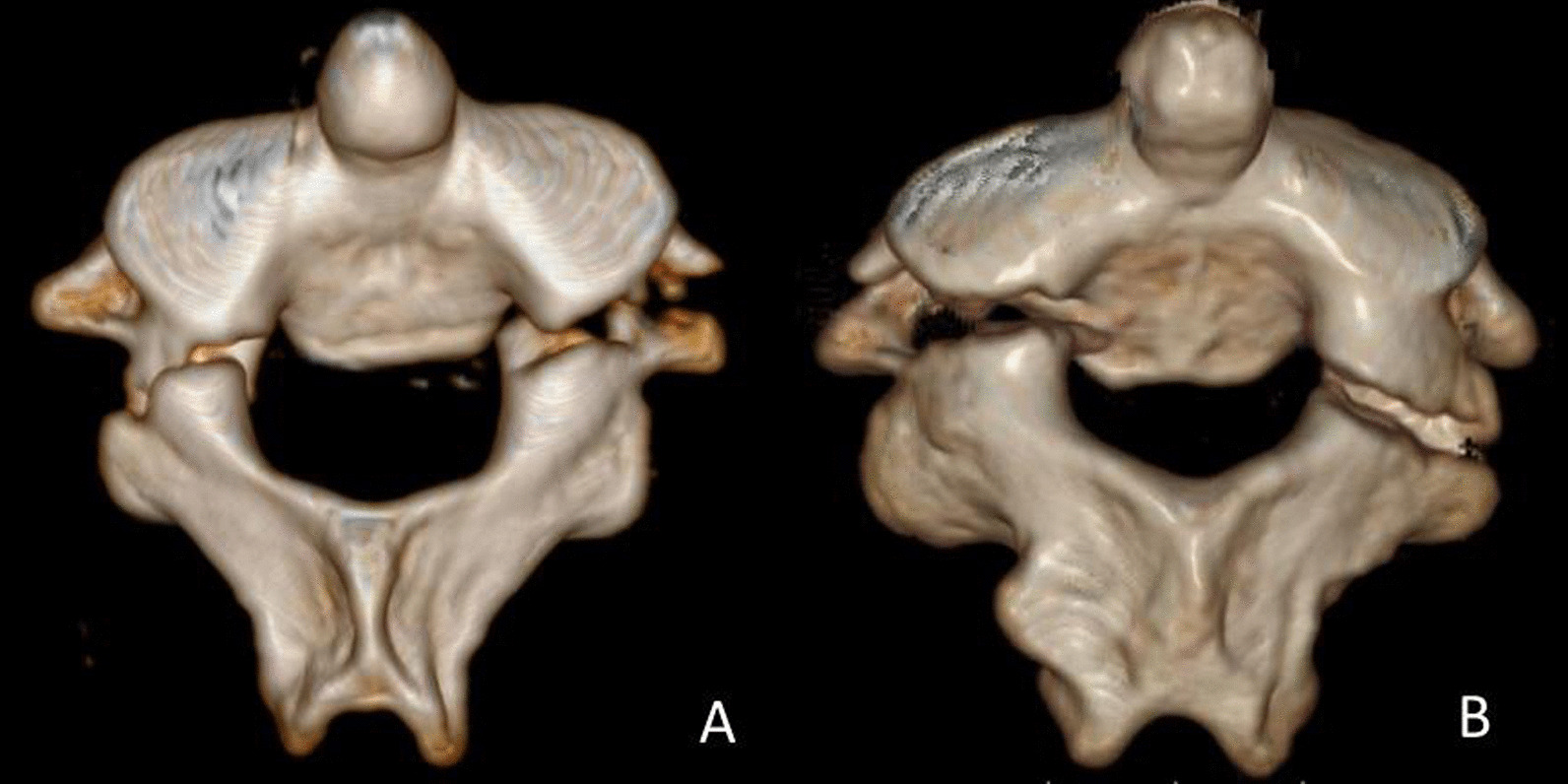
Three-dimensional reconstruction CT image showed injury of middle element on one side and another located in the middle or posterior element on the other side: **A** two middle elements of C2 ring, with bilateral fracture lines located on PIC; **B** middle and posterior elements injuries with left PIC fracture and right inferior articular process fracture

The incidence of various anatomical structures injuries and location of fracture line were observed (Figs. 2 and 3).

## Results

### Anatomical structures involvement in Hangman’s fractures

Total 520 anatomical structures injuries of C2 ring were involved in 210 patients Hangman’s fractures, including 298 facet joints injuries (298/520, 57.3%) and 222 bony injuries (222/520, 42.7%), with 2.5 anatomical structures damaged in connection with C2 ring for each patient.

For 298 facet joints injuries identified in 150 patients, the details of anatomical structures involved were listed as follows:

- 136 superior articular facet injuries in 111 patients (136/520, 26.2%)
- 99 C2-3 disk injuries in 99 patients (99/520, 19.0%)
- 63 inferior articular facet injuries in 63 patients (63/520, 12.1%).

For 222 bony injuries identified in 156 patients:

- 106 PIC injuries in 93 patients (106/520, 20.4%)
- 80 posterior vertebral wall fractures in 78 patients (80/520, 15.4%)
- 36 lamina fractures in 36 patients (36/520, 6.9%).

In the anterior element of axis ring, 315 anatomical structures were involved in 160 patients (315/520, 60.6%), and of which 235 facet joints injuries were identified (235/315, 74.6%), and the remaining was 80 pure bony injuries (80/315, 25.4%). In the middle element of axis ring, 106 anatomical structures were involved in 93 patients (106/520, 20.4%), and all of them were pure bony injuries. In the posterior element, 99 anatomical structures were involved in 99 patients (99/520, 19.0%), and of which 63 facet joints injuries were identified (63/99, 63.6%), and the remaining was 36 pure bony injuries (36/99, 36.4%).

To sum up, facet joints injuries were more common than pure bony injuries (57.3% VS 42.7%) in these 210 patients. Of facet joints injuries, the most common injuries were superior articular facet injuries of C2 (26.2%), followed by C2-C3 disk injuries (19.0%) and inferior articular facet injuries (12.1%). Of pure bony injuries, the most common injuries were PIC fractures (20.4%), followed by fractures of posterior vertebral wall (15.4%) and lamina (6.9%). In regard to different element of C2 ring, the injuries of anterior element (60.6%) were more common than that of middle (20.4%) or posterior (19.0%) element. In the anterior and posterior element of C2 ring, facet joints injuries were more common than pure bony injuries. All of anatomical structure injuries in the middle element were pure bony injuries, due to the only structures in the middle element of C2 ring was PIC.

### The different fracture patterns of Hangman’s fractures

Firstly, 161 (76.7%, 161/210) Hangman’s fractures were asymmetrical, with different elements of axis ring damaged on different sides, and only 49 (23.3%, 49/210) Hangman’s fractures were with the same elements of axis ring; Secondly, typical “Hangman’s fractures” defined as bilateral fractures in the middle element of C2 ring were rare, and the incidence of which was only 6.2% (13/210); Thirdly, when observed with the incidence of different combinations of anterior, middle, and posterior elements, the injuries of anterior element on one side were the most common (178, 84.8%), and the other side injury could be located in the anterior, middle or posterior element (Fig. 2 and Table 1); Finally, injury of middle element of C2 ring on one side with another injury located in the middle or posterior element could be observed (Fig. 3 and Table 1), but there was no Hangman’s fracture with only two posterior element injuries in different sides.

**Table 1:** The incidence of different fracture patterns of Hangman’s fractures

Fracture pattern	Subtype	*N* (%)
*Anterior element*		
Anterior + anterior elements	Fractures of superior articular facet and/or posterior vertebral wall of C2 on different sides	38/18.1
Anterior + middle elements	One fracture of superior articular facet and/or posterior vertebral wall of C2 on one side and another through the C2 PIC on the other side	61/29.0
Anterior + posterior elements	One fracture of superior articular facet and/or posterior vertebral wall of C2 on one side and another through the contralateral inferior articular facet or lamina	79/37.6
*Middle element*		
Middle + middle elements	Fractures PIC of C2 on different sides	13/6.2
Middle + posterior elements	One fracture through C2 PIC on one side and another through the contralateral inferior articular facet or lamina	19/9.0

## Discussion

Since its first description, there was no uniform definition and precise location of Hangman’s fractures [3, 10, 13, 16]. Therefore, investigators reported variable sites and different anatomical locations in Hangman’s fractures [1, 3, 8, 18, 21]. In 2016, Menon et al. [16] conducted a retrospective observational study to provide detailed description of anatomy of the fracture line in Hangman’s fractures, and they found that fracture lines could occur in any part of C2 ring, including pedicles, pars interarticularis, superior or inferior facets, laminae, the posterior vertebral wall, and so on. The anatomical information in Menon et al. [16]’ study may affect the plan of surgical treatment in such injury. However, the limits of Menon et al. [16]’ study were obvious: it focused on motor vehicle accident victims and only include 32 cases; it did not observe the C2-3 disk injuries, which were also an important factor determining the treatment of Hangman’s fractures. Therefore, research addressing the shortcomings of previous study and with large sample or more size of sample is of importance.

To the best of our knowledge, this is the first multiple-center and large-sample study in English providing the detailed description of the fracture line and summarizing the anatomical features of Hangman’s fractures using three-dimensional CT. In addition to the confirming that most of fracture lines in Hangman’s fractures were asymmetrical, and so called “typical hangman’s fractures” described as PIC fractures were rare [1, 10, 14, 22], this study found that facet joints injuries in Hangman’s fractures were more common than pure bony injuries, and superior articular facet injuries were most common. Further, the results of this study demonstrated that: First, when C2 ring was divided into anterior, middle, and posterior elements, the injuries of anterior element were more common than that of middle or posterior element; Second, when observed with the incidence of different combinations of anterior, middle, and posterior elements, the injuries of anterior element on one side were the most common (84.8%), and the other side injury could be located in the anterior, middle or posterior element; Third, injury of middle element of C2 ring on one side with another injury located in the middle or posterior element could be observed, but there was no Hangman’s fracture with only two posterior element injuries.

The clinical implications of this study were listed as follows: 1. In regard to the controversy of term “Hangman’s fractures”, it is not just a truism or just some platitude subject, and an exactly name may help understand the precise anatomical location and pattern of the fracture lines in Hangman’s fracture [1, 3, 5, 21]. Based on the results of this study, we carefully suggest that three-dimensional CT was necessary for such fractures, and using the injuries of precise anatomical structures to replace the general term “Hangman’s fractures” might help assist in planning the reasonable treatment, especially when surgical treatment was used[16, 17]. 2. The results of this current study showed that high prevalence of facet joints injuries in Hangman’s fractures could be observed. Menon et al. [16] also found that facet joint involvement were demonstrated in 50% of their 32 patients, which was consistent with that of our study. The high prevalence of facet joints injuries in such fractures means that most of Hangman’s fractures are involved with intra-articular injuries, which may affect the plan of the suitable treatment [6, 10]. In theory, listhesis of C2 over C3 vertebra is common in unstable Hangman’s fractures, and if a superior facet of axis injury exists, C1-2 joints and C2-3 disk/joints are both unstable [2, 6]. Geol [6] recommended posterior fixation and fusion at C1-3 level should be performed. In our experience, stabilization of both C1-2 and C2-3 joints was necessary if obvious displacement could be observed, and we suggest that posterior fixation and fusion at C2-3 level plus C1-2 temporary fixation might be a proper choice, avoiding to sacrifice atlantoaxial rotation[10].

### Limitations

First, this study is still a retrospective research. Second, fractures extending into the transverse foramen are not recorded and analyzed, because transverse foramen is located outside the superior facet joint and PIC and fracture lines running into this structure are always accompanied with superior facet joint or PIC fractures. Third, CT with three-dimensional reconstructions were the mainly tool in this study, and if MRI, MRA, and CTA could be combined with CT scans, it might give more information about Hangman’s fractures. Last but not least, we did not investigate the fracture type of Hangman's fracture and the mechanism of injury or vertebral instability, and it might provide a clearer clinical message. We hope that these limitations could be resolved in future research.

## Conclusion

In Hangman’s fractures, fracture lines could occur in any part of the C2 ring. Facet joints injuries were more common than pure bony injuries, and the injuries of anterior element were more common than that of middle or posterior element. The high prevalence of facet joints injuries means that most of Hangman’s fractures may be involved with intra-articular injuries. These findings are of clinical significance for describing exactly location and pattern of fracture lines in Hangman’s fractures, thus they may also help in planning the reasonable treatment.
